# Configurational Statistics of Magnetic Bead Detection with Magnetoresistive Sensors

**DOI:** 10.1371/journal.pone.0141115

**Published:** 2015-10-23

**Authors:** Anders Dahl Henriksen, Mikkel Wennemoes Hvitfeld Ley, Henrik Flyvbjerg, Mikkel Fougt Hansen

**Affiliations:** 1 Department of Micro- and Nanotechnology, Technical University of Denmark, DTU Nanotech, Building 345 East, DK-2800 Kongens Lyngby, Denmark; 2 Department of Physics, Technical University of Denmark, DTU Physics, Building 309, DK-2800 Kongens Lyngby, Denmark; Drexel University, UNITED STATES

## Abstract

Magnetic biosensors detect magnetic beads that, mediated by a target, have bound to a functionalized area. This area is often larger than the area of the sensor. Both the sign and magnitude of the average magnetic field experienced by the sensor from a magnetic bead depends on the location of the bead relative to the sensor. Consequently, the signal from multiple beads also depends on their locations. Thus, a given *coverage* of the functionalized area with magnetic beads does not result in a given detector response, except on the average, over many realizations of the same coverage. We present a systematic theoretical analysis of how this location-dependence affects the sensor response. The analysis is done for beads magnetized by a homogeneous in-plane magnetic field. We determine the expected value and standard deviation of the sensor response for a given coverage, as well as the accuracy and precision with which the coverage can be determined from a single sensor measurement. We show that statistical fluctuations between samples may reduce the sensitivity and dynamic range of a sensor significantly when the functionalized area is larger than the sensor area. Hence, the statistics of sampling is essential to sensor design. For illustration, we analyze three important published cases for which statistical fluctuations are dominant, significant, and insignificant, respectively.

## Introduction

In medicine and biology it is often of great interest to quantify the presence of biomolecules accurately. For example, in molecular oncology the ability to detect cancer biomarkers determines how early the disease can be discovered [1].

Magnetic labels used with magnetoresistive sensors offer an alternative to fluorescence detection that may provide higher sensitivity, lower detection thresholds, and wash-free assay protocols [2–4]. Magnetic sensors detect the presence of magnetic beads that are magnetized either by an externally applied field [2–11], by the magnetic field created by the sensor current [12], or by on-chip current lines [13]. As with fluorescent labels, magnetic labels are biologically attached through a sandwich assay to ensure high specificity and affinity. However, the antibody or DNA capture probes often occupy an area larger than the sensor, which is typically a 0.5 − 10 *μ*m wide stripe or an array of such stripes. Consequently, magnetic beads will be randomly distributed both on top of and outside of the sensor structure. However, the signal depends on the bead locations relative to the sensor. For randomly distributed beads, this can cause problems, as the signal from some beads may cancel the signal from others, depending on their relative location [14]. The sensor signal can be described in terms of the degree to which surface is covered by magnetic beads (in short, the *bead coverage*) defined as the projected area of the beads relative to the total area over which the beads are distributed. For a given bead coverage, the sensor signal changes from sample to sample because bead locations differ between samples. These fluctuations in the signal have not been considered in the literature on magnetic biosensors.

Here, we derive the expected value and standard deviation of the signal from a single sensor at fixed coverage and with fluctuations due to statistical sample-to-sample variations in bead locations. The presented approach is generally applicable. The specific condition chosen in this study is magnetic field sensors showing a linear in-plane field response and where the signal is due to magnetic beads magnetized by a homogeneous in-plane magnetic field. These may include, for example, spin-valve giant magnetoresistance sensors [2–9], tunneling magnetoresistance sensors [7] and anisotropic magnetoresistance sensors [11]. We compare these results with the sensor noise characteristics and with limitations due to the discrete nature of magnetic beads. We estimate the lowest detectable sensor coverage and the dynamic range of sensor signals. Finally, we analyze three relevant cases from the literature.

## Theory

### Assumptions and definitions

We assume the magnetic beads to be spherical with radius *R*. Further, we assume that the beads are superparamagnetic with constant magnetic susceptibility *χ* and no magnetic remanence. Moreover, we assume all beads to be magnetized by a homogeneous external field, **H**_ext_, which is applied in the *y*-direction. This field creates a constant magnetic dipole moment, $m_{b}\hat{y}=\frac{4\pi }{3}R^{3}\chi H_{\text{ext}}$, in each bead. Each magnetic bead produces a dipole field that perturbs the external field experienced by the sensor. Later, we will revisit and discuss the validity of these simplifying assumptions.

**Fig 1:**
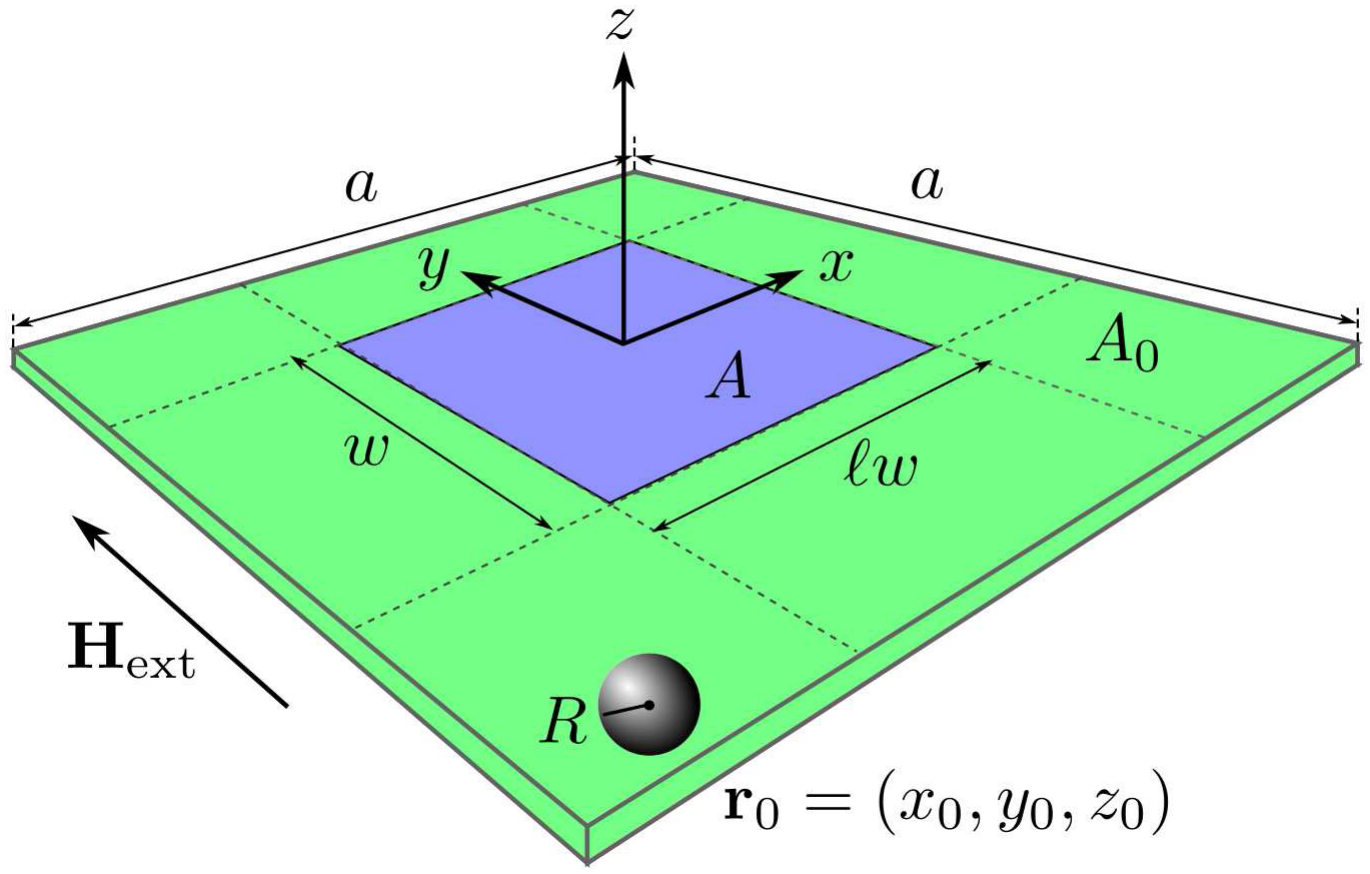
Sensor and functionalized area. Illustration of the sensor area *A* = ℓ*w* × *w* (blue) and functionalized area *A*_0_ = *a* × *a* (blue + green), with definition of geometrical parameters and the coordinate system.

The magnetoresistive sensors used to detect the presence of magnetic beads typically consist of a magnetic stack in a rectangular geometry of area *A* = ℓ*w* × *w*, where ℓ*w* and *w* respectively denote the length and the width of the sensor, and ℓ is the sensor aspect ratio. We choose a coordinate system with origin at the center of the sensor (Fig 1).

We assume that the sensor output voltage (e.g., from a Wheatstone bridge) is proportional to the average *y*-component of the magnetic field, i.e.,

$$\begin{array}{c} V=G\times \frac{1}{A}\int_{A}H_{y}\left(r\right)\;dx\;dy=G\times H_{y}^{\text{avg}}\;, \end{array}$$ (1)

where *G* is the sensitivity. By comparing the sensor output to a reference sensor, the field perturbation from magnetic beads can be separated from the external field. We therefore let $H_{y}^{\text{avg}}$ denote the average magnetic field from only the magnetic beads.

The magnetic beads may attach via specific biological interactions to biomolecules within a functionalized surface area, *A*_0_. We will refer to *A*_0_ as the *functionalized area* and assume that beads are independently distributed, each bead with the same uniform probability density within this area. Unless otherwise specified, we assume that the functionalized area is quadratic and symmetrically placed with respect to the sensor stripe such that *A*_0_ = *a* × *a*. Because of the random distribution of beads, the sensor signal will vary between experiments even if the same number of beads is detected in each experiment. We refer to these random sample-to-sample variations as *configurational fluctuations* or *statistical sampling fluctuations*. In the best case scenario where electrical noise can be neglected, these still produce fluctuations of the sensor output and thereby limit the minimum coverage of magnetic beads that can be assessed reliably from measurements on a single sensor.

In the following analysis, it is convenient to express lengths in units of the sensor width, *w*, and areas in units of *w* ^2^. The resulting dimensionless variables are denoted by tilde, e.g., $\tilde{x}=x/w$ and $\tilde{A}=A/w^{2}$. Moreover, we will define the normalized dimensionless magnetic field as $\tilde{H}=H/\left(\chi H_{\text{ext}}{\tilde{R}}^{3}\right)$.

### Sensor response to single bead

A single homogeneously magnetized bead with center located at ${\tilde{r}}_{0}$ produces a dipole magnetic field at $\tilde{r}$ with *y*-component

$$\begin{array}{c} {\tilde{H}}_{y,1}\left(\tilde{r},{\tilde{r}}_{0}\right)=\frac{1}{3}(\frac{3{\left(\tilde{y}-{\tilde{y}}_{0}\right)}^{2}}{|\tilde{r}-{\tilde{r}}_{0}{|}^{5}}-\frac{1}{|\tilde{r}-{\tilde{r}}_{0}{|}^{3}})\;. \end{array}$$ (2)

The average over the sensor area, $\tilde{A}$, of the dipole field in Eq (2) is

$$\begin{array}{c} \begin{array}{cc} {\tilde{H}}_{y,1}^{\text{avg}}\left({\tilde{r}}_{0}\right)= & I\left({\tilde{x}}_{0}+\frac{\ell }{2},{\tilde{y}}_{0}+\frac{1}{2}\right)-I\left({\tilde{x}}_{0}+\frac{\ell }{2},{\tilde{y}}_{0}-\frac{1}{2}\right)\\ & -I\left({\tilde{x}}_{0}-\frac{\ell }{2},{\tilde{y}}_{0}+\frac{1}{2}\right)+I\left({\tilde{x}}_{0}-\frac{\ell }{2},{\tilde{y}}_{0}-\frac{1}{2}\right)\;, \end{array} \end{array}$$ (3)

where

$$\begin{array}{c} I\left({\tilde{x}}_{0},{\tilde{y}}_{0}\right)=\frac{-{\tilde{x}}_{0}{\tilde{y}}_{0}}{3\left({\tilde{y}}_{0}^{2}+{\tilde{z}}_{0}^{2}\right)\left|{\tilde{r}}_{0}\right|\ell }\;. \end{array}$$ (4)

**Fig 2:**
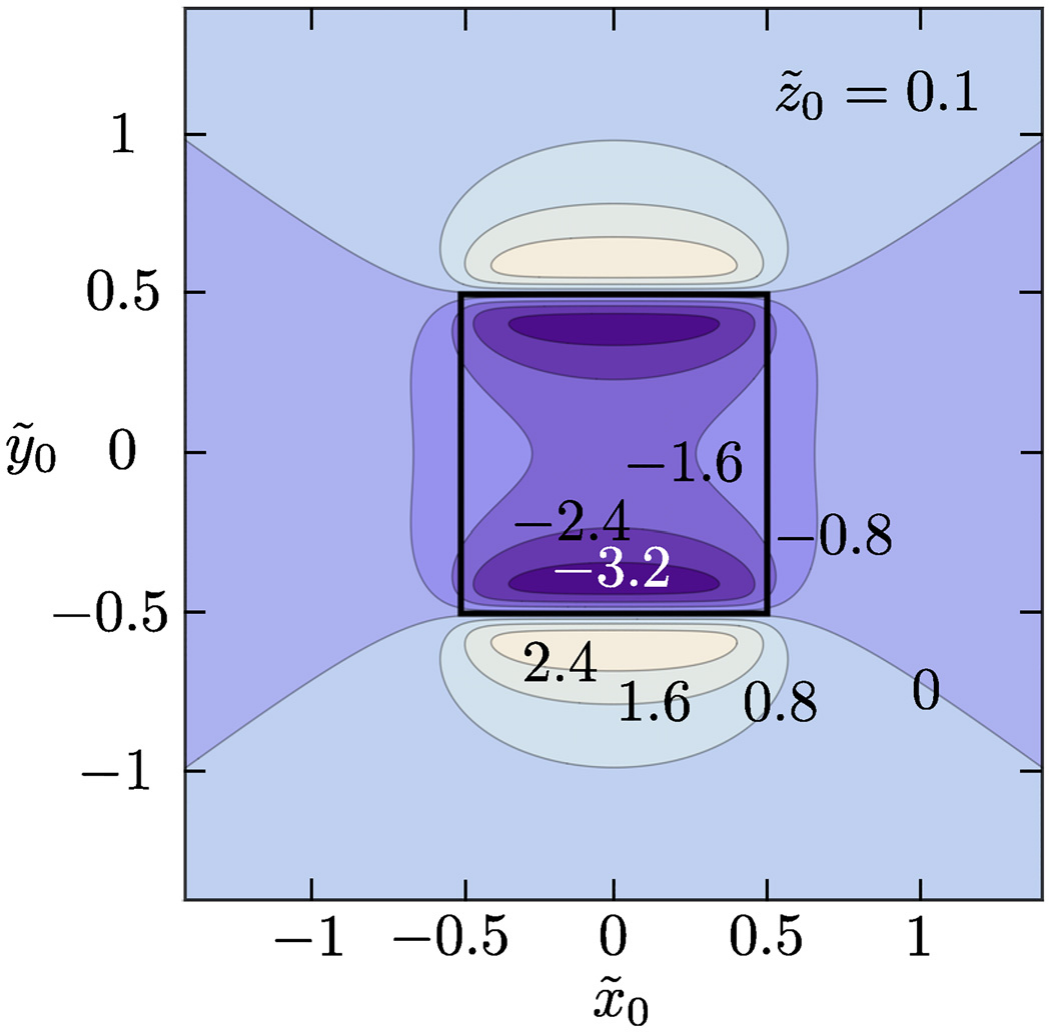
Sensor signal vs. bead position. Contour plots of the normalized sensor signal ${\tilde{H}}_{y}^{\text{avg}}\left({\tilde{r}}_{0}\right)$ as function of bead position ${\tilde{r}}_{0}=\left({\tilde{x}}_{0},{\tilde{y}}_{0},{\tilde{z}}_{0}\right)$ for fixed ${\tilde{z}}_{0}=0.1$. Calculations were carried out for a square sensor (ℓ = 1). Note, that global minima and maxima are found near the sensor edge (black frame) at ${\tilde{y}}_{0}=\pm 0.5$.

The average magnetic field from a single magnetic bead, Eq (3), depends strongly on the bead position **r**_0_ relative to the sensor. This is illustrated for a square sensor in Fig 2, where the signal from a single bead is plotted as function of its position $\left({\tilde{x}}_{0},{\tilde{y}}_{0}\right)$ in the $\tilde{x}\tilde{y}$-plane for ${\tilde{z}}_{0}=0.1$. Depending on whether the bead is over the sensor area or outside the sensor area, a negative or positive sensor output is obtained and the response shows a negative peak when ${\tilde{y}}_{0}=\pm 0.5$ is approached from within the sensor area and a corresponding positive peak just outside the sensor area [14].

Thus, depending on the sensor geometry and the functionalized area, both positive and negative signals are possible. When beads are located both inside and outside the sensor area, we have found that the sensor signal is often dominated by the signal from beads outside the sensor area as these are typically placed at a lower height [15]. Here, we determine the expected value and the standard deviation of the sensor response, taking into account statistical fluctuations. Both of these values are experimentally measurable as estimates based on many repeated experiments with identical sensors (assuming negligible sensor noise) and experimental conditions, as the average sensor response and its sample standard deviation, respectively. More importantly, these parameters are essential to predict the outcome of the individual experiment under the best possible conditions.

Assuming that a single magnetic bead is located with equal probability at any point within the functionalized area, it is convenient to define the configurational expected value of a function $f({\tilde{r}}_{0})$ as

$$\begin{array}{c} E\left[f\right]\equiv \frac{1}{{\tilde{A}}_{0}}\int_{{\tilde{A}}_{0}}f\left({\tilde{r}}_{0}\right)\;d{\tilde{x}}_{0}\;d{\tilde{y}}_{0}\;. \end{array}$$ (5)

Using this notation, the expected value, *S*_1_, of the normalized magnetic field and its variance, $\sigma_{1}^{2}$, for a single bead placed randomly on *A*_0_ are

$$S_{1}=E\left[{\tilde{H}}_{y,1}^{avg}\right]$$ (6)

$$\sigma_{1}^{2}=E\left[{\left({\tilde{H}}_{y,1}^{avg}\right)}^{2}\right]-E{\left[{\tilde{H}}_{y,1}^{avg}\right]}^{2}.$$ (7)

We note that *S*_1_ and *σ*_1_ depend on $\tilde{A}$, ${\tilde{A}}_{0}$ and ${\tilde{z}}_{0}$. Below, to keep the notation simple, general results are expressed in terms of *S*_1_ and *σ*_1_, with their dependence on $\tilde{A}$, ${\tilde{A}}_{0}$ and ${\tilde{z}}_{0}$ understood, whereas their dependence on $\tilde{a}$ and ${\tilde{z}}_{0}$ is explicitly written when a square functionalized area with *A*_0_ = *a* ^2^ is assumed.

### Sensor response to multiple beads

When multiple beads are present, the sensor signal is the sum of their individual contributions. We assume that steric interactions (excluded volume effects) between these beads are negligible. Further, we assume that magnetostatic interactions between the beads and between the beads and the sensor are negligible. Then the beads are independently and identically distributed (*iid*) and the single bead analysis applies to each of these beads. Later, we will revisit and discuss the validity of these simplifying assumptions. Then, the expected value and variance of the summed bead response from *N* beads are

$$S_{N}=E\left[\sum_{i=1}^{N}H_{y,i}^{avg}\right]=NS_{1}$$ (8)

$$\sigma_{N}^{2}=\sigma^{2}\left[\sum_{i=1}^{N}H_{y,i}^{avg}\right]=N\sigma_{1}^{2}.$$ (9)

Often, the exact number of beads is not known, and it is more convenient to describe the response in terms of the bead coverage, *ϕ*. It is the surface area covered by the beads (when projected onto the surface) divided by the total functionalized area,

$$\begin{array}{c} \phi \equiv N\pi R^{2}/A_{0}=N\pi {\tilde{R}}^{2}/{\tilde{a}}^{2}\;. \end{array}$$ (10)

The bead coverage can be estimated independently by, e.g., microscopy, scanning electron microscopy or fluorescence. We note that *ϕ* has a limited range with maximum less than one, because a close-packed monolayer of spheres leaves surface area uncovered,

$$\begin{array}{c} 0\leq \phi \leq \phi_{\text{max}}\equiv \frac{\pi }{2\sqrt{3}}\approx 0.91\;. \end{array}$$ (11)

There is also a lowest non-zero value *ϕ*_1_ for *ϕ*, corresponding to a single bead being present in the functionalized area,

$$\begin{array}{c} \phi_{1}\equiv \frac{\pi {\tilde{R}}^{2}}{{\tilde{a}}^{2}}\;. \end{array}$$ (12)

The value of *ϕ*_1_ also describes the smallest possible increment in *ϕ*, since the bead coverage is incremented by addition of whole beads.

The assumption of iid is justified when the bead coverage is low. A calculation analogous to that leading to Eq (6) in Ref. [16] gives that the assumption of iid beads is a good approximation as long as $\phi <\sqrt{\phi_{1}}/2$. The results for larger values of *ϕ* are only approximative and would be subject to correction terms due to excluded volume effects in a more accurate model.

The expected value and variance of the normalized magnetic field can be written in terms of *ϕ* as

$$S_{\phi }=\frac{\phi A_{0}}{\pi R^{2}}S_{1}=\frac{\phi {\tilde{a}}^{2}}{\pi {\tilde{R}}^{2}}S_{1}\left(\tilde{a},{\tilde{z}}_{0}\right),$$ (13)

$$\sigma_{\phi }^{2}=\frac{\phi A_{0}}{\pi R^{2}}\sigma_{1}^{2}=\frac{\phi {\tilde{a}}^{2}}{\pi {\tilde{R}}^{2}}\sigma_{1}{\left(\tilde{a},{\tilde{z}}_{0}\right)}^{2}.$$ (14)

If *σ*_*ϕ*_ > *S*_*ϕ*_, a single measurement of the bead coverage cannot be trusted. The signal-to-standard deviation ratio (*SDR*) is defined as the expected value of the signal measured in units of its standard deviation,

$$\begin{array}{c} SDR\equiv \frac{|S_{N}|}{\sigma_{N}}=\frac{|S_{1}|}{\sigma_{1}}\sqrt{N}=\frac{|S_{1}\left(\tilde{a},{\tilde{z}}_{0}\right)|}{\sigma_{1}\left(\tilde{a},{\tilde{z}}_{0}\right)}\frac{\tilde{a}}{\tilde{R}}\sqrt{\frac{\phi }{\pi }}\;. \end{array}$$ (15)

Absolute values are used because both positive and negative average values of the signal may occur. Eq (15) accounts for statistical sampling fluctuations in the signal for samples with identical *N* or, equivalently, identical *ϕ*, under the assumption of negligible sensor noise. Since beads were assumed iid, *SDR* is proportional to *N* ^1/2^ or, equivalently, *ϕ* ^1/2^. Note again that *S*_1_ and *σ*_1_ depend on $\tilde{A}$, ${\tilde{A}}_{0}$, and ${\tilde{z}}_{0}$, so the simple prediction above based on iid is valid only when these parameters are fixed.

For convenience below, let *ϕ*_stat_ denote the bead coverage corresponding to *SDR* = 1. Solving Eq (15) with *SDR* = 1, we obtain

$$\begin{array}{c} \phi_{\text{stat}}=\frac{\pi R^{2}}{A_{0}}{\left(\frac{\sigma_{1}}{S_{1}}\right)}^{2}=\pi {\left(\frac{\sigma_{1}\left(\tilde{a},{\tilde{z}}_{0}\right)}{S_{1}\left(\tilde{a},{\tilde{z}}_{0}\right)}\;\frac{\tilde{R}}{\tilde{a}}\right)}^{2}\;. \end{array}$$ (16)

*ϕ*_stat_ is the minimum bead coverage required to ensure a signal of the same magnitude as the statistical sampling fluctuations for a single sampling of the magnetic bead distribution.

Finally, it is also relevant to consider the bead coverage, *ϕ*_noise_, that gives rise to a signal of the same magnitude as the sensor output voltage noise, *V*_noise_. Using the definition of the normalized variables, Eqs (1) and (13), we obtain

$$\begin{array}{c} \phi_{\text{noise}}=\frac{\pi V_{\text{noise}}}{|G\chi H_{\text{ext}}\tilde{R}{\tilde{A}}_{0}S_{1}|}=\frac{\pi V_{\text{noise}}}{|G\chi H_{\text{ext}}\tilde{R}{\tilde{a}}^{2}S_{1}\left(\tilde{a},{\tilde{z}}_{0}\right)|}\;. \end{array}$$ (17)

Note, that *ϕ*_1_, *ϕ*_stat_, and *ϕ*_noise_ may all affect the lower limit of bead coverages that can be resolved. Making the practical simplifying assumption that one of the mechanisms dominates, we can define the resolution as

$$\begin{array}{c} \phi_{\text{res}}\equiv \text{max}\left\{\phi_{1},\phi_{\text{stat}},\phi_{\text{noise}}\right\}\;. \end{array}$$ (18)

Hence, the maximum possible dynamic range (*DR*) for the bead coverage readout is

$$\begin{array}{c} DR\equiv \frac{\phi_{\text{max}}}{\phi_{\text{res}}}=\frac{0.91}{\text{max}\{\phi_{1},\phi_{\text{stat}},\phi_{\text{noise}}\}}\;. \end{array}$$ (19)

Experimentally, the signal for a close-packed magnetic bead monolayer can be approximated by the signal measured when the functionalized area is saturated with magnetic beads. To compare calculations to experiments, it is convenient to express the signal standard deviation, *σ*_*ϕ*_, for a given bead coverage *ϕ* in terms of the saturation (full scale) signal, *S*_*ϕ*_max__. From Eqs (13)–(15), we obtain

$$\begin{array}{c} \frac{\sigma_{\phi }}{S_{\phi_{\text{max}}}}=\sqrt{\frac{\phi }{\phi_{\text{max}}}}\sqrt{\frac{\phi_{\text{stat}}}{\phi_{\text{max}}}}=\sqrt{\frac{S_{\phi }}{S_{\phi_{\text{max}}}}}\sqrt{\frac{\phi_{\text{stat}}}{\phi_{\text{max}}}}\;. \end{array}$$ (20)

The latter factor, $\sqrt{\phi_{\text{stat}}/\phi_{\text{max}}}$, defines the magnitude of the signal standard deviation relative to saturation and the first factor gives the scaling with the bead coverage or the signal with respect to their maximum values.

The above expressions include the discrete nature of the magnetic beads, the position dependence of the signal from magnetic beads, the statistical sampling of a bead distribution, and the sensor noise in the evaluation of the sensitivity and dynamic range of the bead coverage on an area over the sensor. Thus, this provides a much more complete picture of the biosensing capability of a sensor system than considerations of only the sensor noise.

## Results and Discussion

In this section, we first present and discuss the effects of the size of the functionalized area and the bead size. Then we discuss the validity of the simplifying assumptions and finally we apply the developed theory to analyze three important cases found in the literature.

### Impact of size of functionalized area

**Fig 3:**
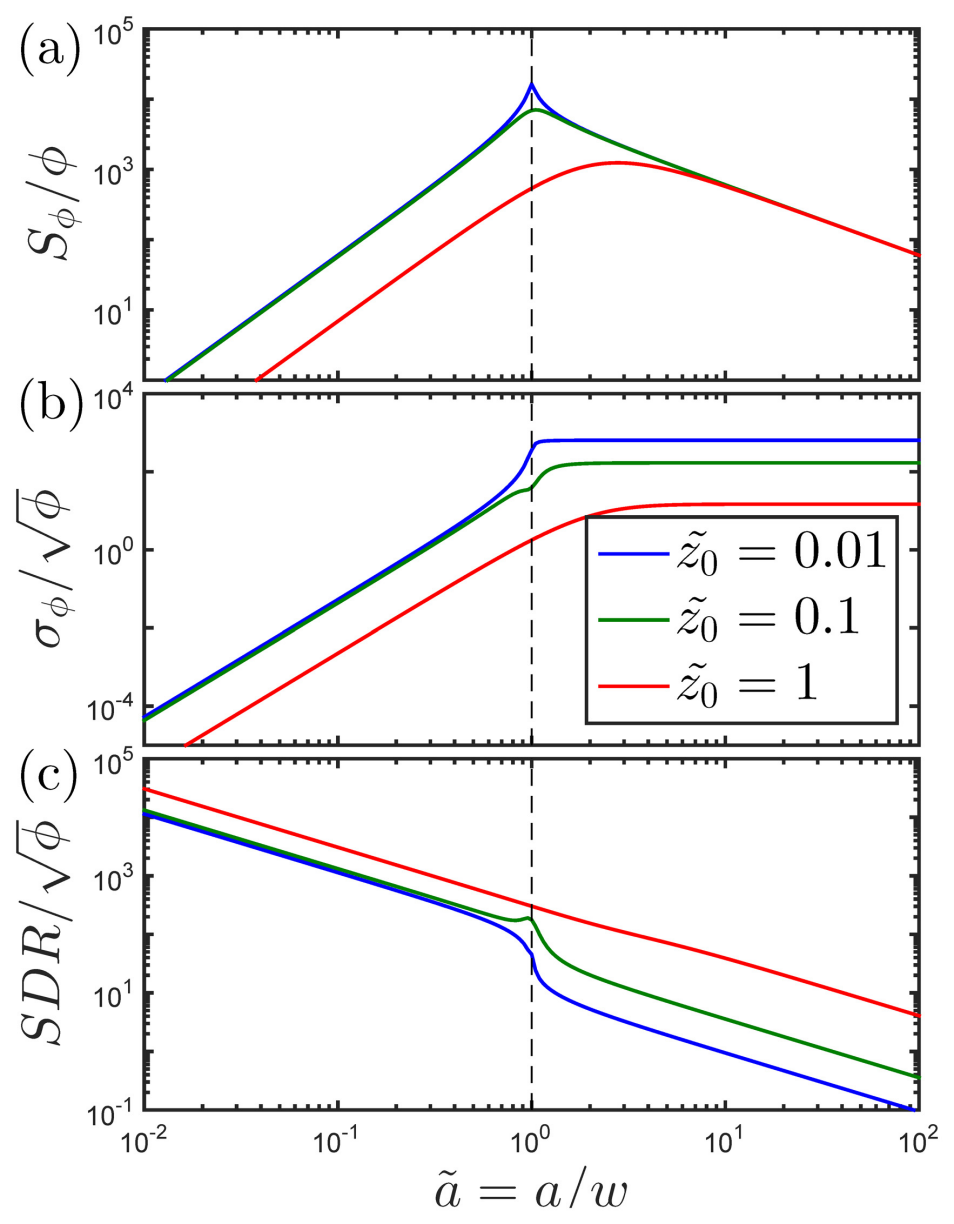
Sensor signal characteristics vs. size of functionalized area. The expected normalized signal as a function of $\tilde{a}$ or *A*_0_ for a constant surface coverage. $\ell =1,\tilde{R}=0.01$.

In this subsection, we study the impact of the size of the functionalized area relative to the sensor area. For simplicity, we consider a square sensor (ℓ = 1) and a square functionalized area of side length *a*. Fig 3 shows (a) *S*_*ϕ*_/*ϕ*, (b) $\sigma_{\phi }/\sqrt{\phi }$, and (c) the corresponding signal-to-standard deviation (*SDR*) vs. $\tilde{a}=a/w$ calculated for the indicated values of ${\tilde{z}}_{0}$ and $\tilde{R}=0.01$.

If the functionalized area is small compared to the sensor size $\left(\tilde{a}\ll 1\right)$, the average magnetic field from a single bead depends only weakly on the bead position within *A*. Moreover, the average bead field, $H_{y,1}^{\text{avg}}\left(r_{0}\right)$ (Fig 2), is well approximated by a constant term plus a small quadratic term in ${\tilde{y}}_{0}$. Upon insertion in Eq (6), the constant term dominates and *S*_1_ is approximately constant. This, combined with Eq (13), gives that *S*_*ϕ*_ is proportional to the area covered by magnetic beads, ${\tilde{a}}^{2}$. Further, upon insertion in Eq (7), the quadratic dependence of $H_{y,1}^{\text{avg}}\left(r_{0}\right)$ on ${\tilde{y}}_{0}$ gives that *σ*_1_ shows a quadratic dependence on $\tilde{a}$. This, combined with Eq (14), gives that *σ*_*ϕ*_ is proportional to ${\tilde{a}}^{3}$. Consequently, *σ*_*ϕ*_ grows faster than *S*_*ϕ*_ for increasing $\tilde{a}$ and the *SDR*-value is proportional to ${\tilde{a}}^{-1}$. The results are observed to be essentially independent of ${\tilde{z}}_{0}$ for $\tilde{a}<1$ when the beads are close to the sensor (${\tilde{z}}_{0}\ll 1$); see coincidence of blue and green curves where $\tilde{a}<1$ in Fig 3.

If the functionalized area is larger than the sensor size ($\tilde{a}>1$), *S*_1_ and *σ*_1_ are proportional to ${\tilde{a}}^{-3}$ and ${\tilde{a}}^{-2}$, respectively. Thus, when the beads are at the same height over and outside the sensor area, *S*_*ϕ*_ is proportional to ${\tilde{a}}^{-1}$ and it decreases to zero for large values of $\tilde{a}$. The value of *σ*_*ϕ*_ approaches a constant, i.e., it becomes independent of $\tilde{a}$ for $\tilde{a}>1$ (Fig 3b). This shows that the statistical signal fluctuation is not reduced by increasing $\tilde{a}$ when $\tilde{a}>1$. When *σ*_*ϕ*_ is constant, the *SDR* is proportional to *S*_*ϕ*_ and thus the *SDR* decreases as ${\tilde{a}}^{-1}$. The value of ${\tilde{z}}_{0}$ only weakly influences the value of *S*_*ϕ*_. However, it has a strong impact on *σ*_*ϕ*_, which increases significantly with ${\tilde{z}}_{0}$ at low values of ${\tilde{z}}_{0}$. This is due to the increasing magnitude of the sensitivity peaks in the sensor response near the sensor edge at ${\tilde{y}}_{0}=\pm 0.5$ for low ${\tilde{z}}_{0}$.

The results clearly indicate that for this geometry of the functionalized area and a fixed value of ${\tilde{z}}_{0}$, it is beneficial to restrict the beads to a central area on top of and within the sensor area. The optimum choice of the size of this area depends on the magnetic field noise of the sensor. The size of the functionalized area should be large enough to support a resolution of *ϕ* and a dynamic range matching assay requirements (Eq (19)). Moreover, the above considerations indicate that for small values of ${\tilde{z}}_{0}$, it may be advantageous to avoid using the area near the sensor edge at ${\tilde{y}}_{0}=\pm 0.5$, since beads located in this area give rise to a high signal, but also to an even higher statistical fluctuation of the signal.

### Impact of magnetic bead size

**Fig 4:**
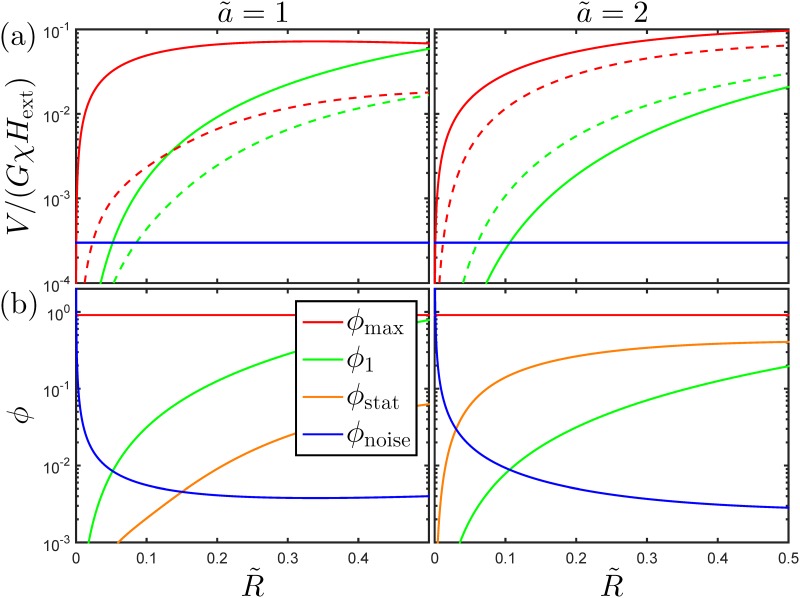
Characteristics of signal and bead coverage vs. bead size. (a) The normalized sensor signal and statistical standard deviation for the maximum surface coverage (solid and dashed red lines) and for a single bead (solid and dashed green lines) along with a sensor noise level chosen to *V*_noise_/(*GχH*_ext_) = 3 ⋅ 10^−4^ (blue line). (b) Values of *ϕ*_max_ (red line), *ϕ*_1_ (green line), *ϕ*_stat_ (orange line) and *ϕ*_noise_ (blue line). The calculations were performed for square sensors (ℓ = 1), the indicated values of $\tilde{a}$, and $\tilde{h}=0.05$.

In this section, we study the impact of the bead size on the sensor signal and on the range of values of *ϕ* that can be reliably quantified experimentally. For simplicity, we consider a square sensor (ℓ = 1). We assume that the sensor area is given a protective coating of thickness *h*, such that the height of a magnetic bead over the sensor is given by *z*_0_ = *h* + *R*. We will consider square bead functionalized areas with *a* = *w* ($\tilde{a}=1$) and *a* = 2*w* ($\tilde{a}=2$), respectively. Fig 4a shows the dimensionless normalized sensor signal $V/\left(G\chi H_{\text{ext}}\right)={\tilde{R}}^{3}S_{\phi }$ for *ϕ* = *ϕ*_max_ and *ϕ* = *ϕ*_1_, as function of the normalized bead radius $\tilde{R}$ for $\tilde{h}=0.05$. The horizontal line in the figure indicates the normalized sensor noise level, chosen for illustrative purposes to *V*_noise_/(*GχH*_ext_) = 3 × 10^−4^. Fig 4b shows the corresponding values of *ϕ*.

The normalized sensor signal for a close-packed monolayer (*ϕ* = *ϕ*_max_, red lines) in Fig 4a first increases sharply with increasing $\tilde{R}$ and then assumes an approximately constant value. This response to an increase in $\tilde{R}$ results from an increase in the magnetic volume, which increases the signal, and a decrease of the sensitivity near the sensor edges at ${\tilde{y}}_{0}=\pm 0.5$ due to the increase in ${\tilde{z}}_{0}$. When $\tilde{R}$ is low, the number of beads is correspondingly higher, and therefore the increase of signal for *ϕ* = *ϕ*_max_ is significantly steeper than that observed for a single magnetic bead (*ϕ* = *ϕ*_1_). For $\tilde{a}=1$, the signal is generated by a single magnetic bead when $\tilde{R}\approx 0.5$. For $\tilde{a}=2$, the signal has both positive and negative contributions (Fig 2). Increasing values of $\tilde{R}$ modify the sensitivities near all sensor edges. As a result, the overall signal for $\tilde{a}=2$ becomes slightly larger than for $\tilde{a}=1$. For higher values of $\tilde{a}$, however, we observe that the signal decreases towards zero as expected (data not shown). For sensors with a larger aspect ratio (ℓ > 1), the sensor edges at ${\tilde{x}}_{0}=\pm \ell /2$ are of smaller relative importance, and we generally expect a decrease of the signal magnitude for a fixed value of ${\tilde{z}}_{0}$ when $\tilde{a}>1$. We have studied the effect of the thickness $\tilde{h}$ of the sensor coating and found it to influence the result only weakly when $\tilde{h}\ll 1$. From Fig 4a, we note for a single bead, that the signal standard deviation is smaller than the signal for $\tilde{a}=1$, but larger than the signal for $\tilde{a}=2$.

Expressed in terms of the degree of coverage, *ϕ*, Fig 4b shows that both *ϕ*_1_ and *ϕ*_stat_ increase slowly with increasing $\tilde{R}$. For $\tilde{a}=1$, *ϕ*_1_ is about an order of magnitude larger than *ϕ*_stat_ for all values of $\tilde{R}$ and thus dominates *ϕ*_res_. Hence, for this case, the statistical sampling fluctuations are not important. However, for $\tilde{a}=2$, we find that *ϕ*_res_ is dominated by *ϕ*_stat_ and that the dynamic range is significantly reduced. The value of *ϕ*_noise_ is found to diverge for $\tilde{R}\to 0$ due to the signal decrease in this limit, and it becomes essentially constant for higher values of $\tilde{R}$. Note, that *ϕ*_noise_ depends on the size of the functionalized area (Eq (17)).

#### How to optimize the choice of bead size

The above considerations show how the magnetic bead size can be chosen to optimize the magnetic biosensing readout to obtain the lowest limit of detection (LOD) or to have the largest dynamic range. The lowest LOD is obtained when *ϕ*_1_ is significantly larger than both *ϕ*_noise_ and *ϕ*_stat_, as the sensor then can detect the binding of a single magnetic bead. We note for the two values of $\tilde{a}$ investigated that *ϕ*_1_ > *ϕ*_stat_ only occurs for $\tilde{a}=1$, and hence that it is difficult for $\tilde{a}=2$ to identify a unique condition with the lowest LOD. For $\tilde{a}=1$, it should be noted that once the bead size is above the limit where a single bead can be detected, only little can be gained by a further increase of $\tilde{R}$ towards 0.5. On the contrary, the dynamic range decreases significantly and approaches a value of 1, and this may make it difficult to assess and resolve the level of unspecific binding of beads to a negative control sensor.

The considerations also show that for given sensor parameters, it is possible to identify an operating condition that maximizes the dynamic range in *ϕ* and hence the dynamic range for the biosensor. This is obtained for the value of $\tilde{R}$ corresponding to the minimum value of *ϕ*_res_ obtained as the value of *ϕ* where *ϕ*_noise_ = max{*ϕ*_1_, *ϕ*_stat_}. For the parameters used in Fig 4, this is observed for $\tilde{R}\approx 0.05$ ($\tilde{a}=1$) and for $\tilde{R}\approx 0.03$ ($\tilde{a}=2$), corresponding to dynamic ranges of 2.0 and 1.5 orders of magnitude, respectively. Note that the value of *ϕ*_noise_ also depends on the sensor operation conditions, i.e., on the excitation field, *H*_ext_. Also note that for real sensors, there may be a height difference between the magnetic beads on top of the sensor area and outside the sensor area, which will modify the behavior compared to the results above [15]. Finally, note that the above considerations do not include the diffusion and binding kinetics of the beads to the functionalized area, which may affect the time to reach equilibrium and the stability of the result.

### Validity of simplifying assumptions

The results presented above were based on a number of simplifying assumptions. Here, we discuss the validity of these assumptions and the anticipated consequences of relaxing them.

#### Magnetic bead properties

We assumed that the beads have a constant magnetic susceptibility. Generally, the magnetic field sensors are operated at low magnetic fields (< 5 mT) to ensure that the sensors operate in their linear regime and to reduce constraints on the electromagnets producing the magnetic excitation field. Generally, magnetic beads and nanoparticles show very small deviations from linear behavior for such small magnetic fields (see, e.g., [17]), and therefore the assumption of constant magnetic susceptibility is justified in most cases. A realistic sample of magnetic beads also displays a distribution of bead sizes and magnetic moments. Above, we found that the dependence on the magnetic bead position easily can cause substantial signal variation. We therefore expect that this effect usually will dominate over the effect of a distribution of magnetic bead properties, unless the dispersion of bead properties is extreme. However, if a system geometry is applied for which the signal depends only weakly on bead positions (e.g., if beads are placed only on the central area of a sensor), then it may be relevant to account also for a dispersion in bead properties in the statistical analysis of the sensor signal.

#### Magnetic interactions between beads

In the analysis above, we neglected dipole interactions between magnetic beads. These interactions may modify the magnetic response of the beads as well as their distribution. Hence it is relevant to discuss the validity of this assumption in some detail. The magnetic dipole interaction between two magnetic beads can be considered negligible when the magnetic dipole field from a bead is much smaller than the external magnetizing field, **H**. Following the analysis of [14], this assumption corresponds to the criterion $\chi R^{3}/\left(3r_{\text{bb}}^{3}\right)\ll 1$, where *r*_bb_ is the center-to-center separation of two beads. Due to demagnetization effects, the upper limit of the magnetic susceptibility for a homogeneously magnetized sphere is *χ* = 3. Thus, we can reduce the criterion to $R^{3}\ll r_{\text{bb}}^{3}$. As *r*_bb_ ≥ 2*R*, this criterion is seen to be fulfilled for all cases but the most dense packing of magnetic beads. When magnetic interactions are significant, the effective magnetic susceptibility of an ensemble of beads may be larger than that based on non-interacting beads and therefore a dense packing of beads will give a correction to the signal calculated assuming non-interacting beads. In that case our predictions are only estimates, lower bounds on the true results.

#### Magnetostatic field from sensor structure

In the analysis above, we also neglected the influence of the magnetostatic field due to the magnetic layers of the sensor structure. The magnetic field close to the surface of a structure that is magnetized along the external magnetic field, will be parallel to the external field outside the structure and antiparallel to the external field on top of the structure. Consequently, the contribution to the sensor signal from beads magnetized by the magnetostatic field will generally not suffer from the cancellation effects observed for a homogeneous magnetic field [9]. Therefore, as the signals are superposed, a signal may be observed even when the signal due to beads magnetized by the homogeneous magnetic field is zero. In our calculations, we have assumed that the signal due to the external magnetic field dominates. The signal due to the magnetostatic field from the sensor depends highly on the detailed sensor design and operation and is thus not easy to include in our general considerations. For specific sensor designs and operating conditions, we recommend including these in the signal estimates when relevant. The two contributions to the signal can be superposed to find the total signal. The variance of the total signal is more complicated to calculate, however, because the two contributions are correlated. Therefore, variances of the individual signals do not add up to the variance of the total signal sum, $\sigma_{1}^{2}$, which must be calculated for the combined signal. We emphasize that even if the signal itself is dominated by the magnetostatic field contribution (e.g., if contributions due to beads magnetized by the homogeneous external magnetic field mostly cancel each other), the *fluctuations* in the signal due to the external magnetic field cannot be neglected. Due to the strong position-dependence, these are likely to dominate the statistical fluctuations of the total sensor signal.

#### Bead probability distribution

We also assumed that the beads are independently and identically distributed. We have already discussed the validity and impact of this assumption in the theory section on the Sensor response to multiple beads. Finally, we assumed that the beads are uniformly distributed. This assumption is plausible and the simplest possible case. This simplicity simplified our presentation and derivations above. It is easily repeated for a more general assumption about the distribution of beads. A non-uniform distribution could result from the protocol of the assay. For example, a magnetic-field-accelerated incubation may enhance the probability of beads binding at certain locations. As long as the beads are independently distributed, our calculation of the statistical variance of the signal remains valid with little change. One just cannot characterize the bead distribution fully using the constant magnetic bead coverage that we introduced for a uniform distribution. However, the signal and its fluctuations are dominated by contributions from areas near the sensor edge at ${\tilde{y}}_{0}=\pm 0.5$. Therefore, even for a non-uniform probability distribution for the magnetic beads, the results obtained for the uniform case may have a high predictive value for the relative importance of the statistical fluctuations in the signal, as long as the uniform bead coverage matches the actual density of beads near the edge of the sensor.

## Case Studies

In this section, we use the theoretical framework from the theory to analyze the signal and dynamic range in terms of our simple presented model for three quite different GMR-based magnetic biosensors from the literature, presented by Graham *et al*. [5], Martins *et al*. [6], and Gaster *et al*. [2]. Details on the geometrical parameters of the sensors, including the height difference between beads on the sensor area and outside the sensor area, are given in the Supplementary Material, Section S1 in S1 Table.

Graham *et al*. [5] use a 6 × 2 *μ*m^2^ sensor stripe to detect the DNA-mediated binding of 250 nm magnetic beads. They employ tapered current lines to attract and focus the magnetic beads onto an area on and near the sensor stripe. For simplicity, we approximate the functionalized area with a square centered with respect to the sensor stripe and with edge length *a* = 16 *μ*m, corresponding to the length of the tapered part of the current line. The authors investigate experimentally (in singlet) the sensor response to a series of three subsequent exposures to a suspension of DNA-functionalized magnetic beads and correlate the sensor response to the number of magnetic beads on the sensor stripe observed in a microscope. They find a linear correlation between the number of magnetic beads and the sensor response and conclude that concentrations in the 10 fM range, corresponding to the binding of a single magnetic bead, may be within reach.

Martins *et al*. [6] is a later study by the same group, in which they use a U-shaped sensor stripe with total dimensions 80 × 2.5 *μ*m^2^ and a U-branch length of 40 *μ*m to detect the DNA-mediated binding of 250 nm magnetic beads. The sensor stripe is placed inside a U-shaped current line structure used to attract and focus the magnetic beads to the sensor stripe. The sensor stripe is covered by a patch of gold (43 × 13 *μ*m^2^), which is selectively coated with capture DNA to define the functionalized area. The authors study the response vs. DNA concentration and conclude that, using field-assisted hybridization, they can detect concentrations from 1 fM to 10 pM corresponding to normalized signals ranging from 0.005 (1 fM) to 0.05 (saturation). Error bars of about 5% (1 fM) to 10% (saturation) of the saturation signal are reported in their dose-response curve. Values were obtained from measurements on at least five sensors.

Gaster *et al*. [2] use a meandering sensor with 32 series-connected stripes of dimensions 100 × 0.75 *μ*m^2^ to detect the binding of 50 nm magnetic beads in a sandwich assay for protein detection. The functionalized area, defined by spotting of capture antibodies, is significantly larger than the extent of the meandering sensor. For simplicity, we therefore assume this area to be infinite. The authors study the response vs. antigen concentration and conclude that they can detect and discriminate between concentrations in the range from 1 fM to 5 nM, corresponding to rms signals ranging from 1 *μ*V to 200 *μ*V. Error bars corresponding to *relative* signal standard deviations of a few percent are reported. Values were obtained from measurements on at least four nominally identical sensors on the same sensor chip.

Table 1 states the values of *ϕ*_1_, *ϕ*_stat_, log_10_(*DR*) and *S*_*ϕ*_max__ calculated from the geometrical parameters given in S1 Table for the three cases. The results are given for the idealized case where magnetic beads are only present on top of the magnetic sensor (first row) as well as for the more realistic case where the functionalized area extends outside the sensor area as described above (second row). The values of *ϕ*_noise_ could not be assessed from the information presented in the case studies.

**Table 1:** Sensor signal characteristics when the functionalized area is identical to the sensor area and larger than the sensor area. Values of *ϕ*_1_, *ϕ*_stat_, log_10_(*DR*), and *S*_*ϕ*_max__ calculated from the geometrical parameters given in S1 Table for the indicated cases from the literature and assuming independently and identically distributed magnetic beads with an average bead surface coverage of *ϕ*. The top row for each case assumes that magnetic beads are only present on top of the sensor area. The bottom row for each case assumes functionalized areas extending outside the sensor area as described in the text.

	*ϕ*_1_ [%]	*ϕ*_stat_ [%]	log_10_(*DR*)	*S*_*ϕ*_max__
Graham *et al*. [5]	0.4	2.3 ⋅ 10^−2^	2.4	-147
	1.2 ⋅ 10^−2^	2.3 ⋅ 10^3^	-1.4	-2
Martins *et al*. [6]	2.5 ⋅ 10^−2^	1.0 ⋅ 10^−3^	3.6	-253
	8.8 ⋅ 10^−3^	7.5	1.1	9
Gaster *et al*. [2]	8.2 ⋅ 10^−5^	5.6 ⋅ 10^−6^	6.1	-686
	0	7.2 ⋅ 10^−4^	5.1	705

For all three cases, we find for the idealized case (top rows) that *ϕ*_res_ is limited by the discrete counting of individual beads—i.e., by *ϕ*_1_—under the assumption that the sensor noise is negligible. We estimate values of the dynamic range to be 2.4 to 6 orders of magnitude. However, when the beads are distributed on a more realistic functionalized area (bottom rows), the results are dramatically different.

For Graham *et al*. [5], we find that a monolayer of magnetic beads gives rise to nearly zero value of *S*_*ϕ*_max__ and that *ϕ*_stat_ ≫ *ϕ*_max_. Hence, the analysis predicts that statistical sampling fluctuations prevent the assessment of the true average value of *ϕ* from a single sensor measurement. The assumption of iid magnetic beads may be an oversimplification, but the above overall conclusion that poor statistical sampling prevents general assessment of *ϕ* from the measurement on a single sensor chip remains valid even for a smaller functionalized area as the signal fluctuations are dominated from contributions near the sensor edges. Due to the large value of *ϕ*_stat_, a realistic increase of the number of sensors will therefore not significantly improve the situation. A lowering of the relative importance of the statistical fluctuation could be achieved by increasing the sensor area and by limiting magnetic beads to be bound either inside or outside the sensor area.

For Martins *et al*. [6], a monolayer of beads still gives rise to nearly zero signal, and the positive value of *S*_*ϕ*_max__ indicates that the signal is weakly dominated by contributions from magnetic beads outside the sensor area. Inserting *ϕ*_stat_ in Eq (20) yields a relative statistical sampling fluctuation at saturation for a single sensor of 29%. Assuming that five sensors were used to determine the values presented by the authors, the corresponding relative standard deviation on the mean is 13%. For the lowest DNA concentration (1 fM), the authors obtain a value of about 0.005 compared to a saturation value of 0.05. Using Eq (20), we estimate a standard deviation relative to saturation of 4%. Both values compare quite well with the respective error bars of about 10% and 5% of the saturation value presented by the authors. Based on our analysis, we conclude that the resolution of this sensor design still is likely to be limited by statistical sampling fluctuations. The relative importance of the fluctuations could be reduced by limiting the beads to be bound either inside or outside the sensor area.

For Gaster *et al*. [2], the signal is clearly dominated by the magnetic beads outside the sensor area, which give rise to a positive value of *S*_*ϕ*_max__. Due to the large aspect ratio of the sensor and the small magnetic bead size, our calculations predict that the statistical sampling fluctuations are very small (*ϕ*_stat_ ≪ *ϕ*_max_) and hence that the sensor signal, assuming negligible noise, has a dynamic range of up to five orders of magnitude. Experimentally, the authors find quite small standard deviations in their dose-response curve, which covers 2.2 orders of magnitude in the signal. According to the considerations above, statistical sampling fluctuations are negligible on this scale, so signal variations are likely due to sensor noise and/or variations in experimental conditions between experiments.

## Conclusion

We have described the statistics of the magnetoresistive sensor signal as function of the functionalized area for beads magnetized by a homogeneous in-plane magnetic field. We have presented a general theoretical framework to (1) estimate the relative importance of sample-to-sample fluctuations of the sensor signal from a finite number of beads, and (2) assess the ability to determine a reliable value of the bead coverage from a single sensor measurement.

We have exemplified and discussed the signal from of a square sensor with a square functionalized area centered on the sensor area. We have studied the effect of the size of the functionalized area for a fixed size of magnetic beads. The signal level grows with the size of the functionalized area until it covers the sensor area, after which the signal decreases. The statistical sampling fluctuations of the signal increase with the size of the functionalized area (faster than the signal) and remain constant when the functionalized area extends beyond the sensor area. We also investigated the effect of the size of the magnetic bead for two sizes of the functionalized area. The statistical sampling fluctuations are found to be unimportant when the magnetic beads are limited to the sensor area, and the bead size can be chosen to maximize the dynamic range of the sensor signal or to obtain maximum sensitivity to the binding of a single binding event. When the functionalized area is allowed to extend beyond the sensor area such that beads contribute both positively and negatively to the sensor signal, the picture changes dramatically and statistical sampling fluctuations are likely to play a key role for the sensor signal fluctuations and the resulting obtainable dynamic range. Further, we have discussed limitations imposed by the simplifying assumptions and indicated how the effect of, e.g., the magnetostatic field of the sensor can be considered for a more detailed analysis of specific sensor designs.

Finally, we have used the theoretical framework to analyze three cases from the literature. For Graham *et al*. [5], we found that the statistical sampling fluctuations are prohibitively large and thus that the sensor system design is not feasible for accurate assessment of the bead coverage. For Martins *et al*. [6], we found that statistical sampling fluctuations are significant and limit the ability to make accurate determinations of the bead coverage. For Gaster *et al*. [2], we found that the statistical sampling fluctuations are so small that they are unimportant and hence that the ability of the sensor to make accurate estimates of the bead coverage is limited by other factors. The results of the theoretical analysis are, at least qualitatively, in agreement with the experimental results for the three cases. It would be interesting to compare the theoretical predictions to systematic experiments on a large number of magnetic sensors of different designs, using different operating conditions with nominally identical bead coverages to test the limits of the predictions. Unfortunately such experiments are not yet available in the literature.

The results demonstrate how the presented theory can be used to take all relevant sensor parameters into account—including the sensor noise, the statistical sample-to-sample fluctuations and the discrete nature of the magnetic beads—to assess the applicability of a sensor design and operation. We note that the presented general theoretical framework can be modified to deal with other experimental geometries and field excitation schemes, for example, where magnetic beads are magnetized using the field from the sensor bias current [12] or on-chip current lines [13]. Further, we have indicated how the effect of the magnetostatic field of the sensor can be included in a future expansion of the model.

Future work involves using the framework to optimize operating conditions (magnetic bead size and magnetic field) to maximize performance of existing designs and to compare to more elaborate experimental studies.

## Supporting Information

S1 TableGeometrical parameters for the case studies.Dimensions of the GMR sensor and magnetic beads used in the indicated literature case studies. The sensors have a width *w* and a total length ℓ*w*. *R* denotes the radius of the magnetic beads used in the studies. *z*_0_ = *h* + *R* and $z_{0}^{\text{out}}=h^{\text{out}}+R$ denote the bead center to sensor layer distance for beads on top of the sensor area and outside the sensor area, respectively.(PDF)
