# Supplementary material for: Configurational Statistics of Magnetic Bead Detection with Magnetoresistive Sensors
Source: PLoS One. 2015 Oct 23;10(10):e0141115. doi: 10.1371/journal.pone.0141115 (PMC4619777; doi:10.1371/journal.pone.0141115)
Supplement: S1 Table — Dimensions of the GMR sensor and magnetic beads used in the indicated literature case studies. The sensors have a width w and a total length ℓw. R denotes the radius of the magnetic beads used in the studies. z 0 = h + R and z0out=hout+R denote the bead center to sensor layer distance for beads on top of the sensor area and outside the sensor area, respectively. (PDF) [file pone.0141115.s001.pdf]

Supplementary Information

Configurational statistics of magnetic bead detection with magnetoresistive sensors

Anders Dahl Henriksen[1], Mikkel Wennemoes Hvitfeld Ley[2], Henrik Flyvbjerg[1], and Mikkel Fougt Hansen[1,*]

[1]Department of Micro- and Nanotechnology, Technical University of Denmark, DTU Nanotech, Building 345 East, DK-2800 Kongens Lyngby, Denmark
[2]Department of Physics, Technical University of Denmark, DTU Physics, Building 309, DK-2800 Kongens Lyngby, Denmark
[*]Mikkel.Hansen@nanotech.dtu.dk

S1 Geometrical parameters of sensors

Three GMR sensors were investigated as case studies. In the stack compositions below, all thicknesses are given in nanometer and the free layer of the stack is underlined.

Graham *et al.*[1] used a rectangular sensor of length $\ell w = 6$ µm and width $= 2$ µm. The spin valve sensor stripes had a nominal magnetic stack of Ta(2)/NiFe(3)/CoFe(2.5)/Cu(2.6)/CoFe(2.5)/MnIr(6)/Ta(3)/TiW(N)(15) and a total thickness of 37 nm. Further, the sensor and its surroundings were passivated with a 200 nm thick SiO_2 layer. The magnetic stack was structured using ion milling with an overetch of 5 nm.[?] The height from the sensitive layer (taken to be in the middle of the two sensitive layers) to the top of the sensor surface was $h = (2.75+2.6+2.5+6+3+15+200)$ nm$\approx$ 232 nm and outside the sensor surface, the height was $h^{\text{out}} \approx (232-37-5)$ nm= 190 nm. Nanomag®-D magnetic particles with a diameter of $2R = 250$ nm (Micromod, Germany) were used for the experiments.

Martins *et al.*[2] used a U-shaped sensor of a total length of $\ell w = 80$ µm and width $w = 2.5$ µm. The multilayer spin-valve stack consisted of Ta(1.5)/NiFe(3)/CoFe(2.5)/Cu(2.1)/CoFe(2.5)/MnIr(8)/Ta(2)/TiW(N)(15) and a total thickness of 37 nm. The sensor and its surroundings were coated with a double oxide layer of Al_2O_3 (100 nm)/SiO_2 (200 nm). The magnetic stack was structured using ion milling with an overetch of 5 nm.[?] Further, a 43×13 μm Ti(5 nm)/Au(20 nm) pad centered on top of the sensor U-branch was U-branch area for selective functionalization with DNA via thiol chemistry. The height from the sensitive layer (taken to be in the middle of the two sensitive layers) to the top of the sensor surface was $h = (2.75+2.1+2.5+8+2+15+300+25)$ nm$\approx$ 357 nm and outside the sensor surface, the height was $h^{\text{out}} \approx (357-37-5)$ nm= 315 nm on the gold patch. Nanomag®-D magnetic particles with a diameter of $2R = 250$ nm (Micromod, Germany) were used for the experiments.

Gaster *et al.*[3] used a meandering design with 32 sensor stripes with a spacing of $s = 0.75$ µm, each having a length of $\ell w =$ 100 µm and a width $w = 0.75$ µm. The sensor used a magnetic stack of Ta(5)/Seed layer(4)/MnIr(8)/CoFe(2)/Ru(0.8)/CoFe(2)/Cu(2.3)/CoFe(1.5)/Ta(3), which had a total thickness of 29 nm. The magnetic stack was structured using ion milling resulting in an overetch. This stack and its surroundings were further coated with a protective oxide of SiO_2(10)/Si_3Ni_4(20)/SiO_2(10).[4] The height difference between the stack and its surroundings was measured postdeposition using an atomic force microscope and determined to 60 nm.[5] This height difference includes the thickness of the stack and the overetching during ion milling. The height from the sensitive layer to the top of the sensor surface was $h = (0.75+3+40)$ nm$\approx$ 44 nm and outside the sensor surface, the height was $h^{\text{out}} \approx (44-60)$ nm= -16 nm. Microbeads from Miltenyi Biotech with a nominal diameter of $2R = 50$ nm were used as magnetic tags.

All of the above sensors were configured to be sensitive in the y-direction (perpendicular to the long axis of the sensors).

The geometrical parameters used in the calculations are summarized in Table S1

Table S1. Geometrical parameters for the case studies. Dimensions of the GMR sensor and magnetic beads used in the indicated literature case studies. The sensors have a width w and a total length ℓw. R denotes the radius of the magnetic beads used in the studies. $z_0 = h + R$ and $z_0^{\text{out}} = h^{\text{out}} + R$ denote the bead center to sensor layer distance for beads on top of the sensor area and outside the sensor area, respectively.

	w [μm]	ℓw [μm]	z_0 [μm]	z_0^{out} [nm]	R [nm]
Graham *et al.*[1]	2	6	357	315	125
Martins *et al.*[2]	2.5	80	482	430	125
Gaster *et al.*[3]	0.75	32×100	69	9	25

References

1. Graham, D. *et al.* Magnetic field-assisted DNA hybridisation and simultaneous detection using micron-sized spin-valve sensors and magnetic nanoparticles. *Sens. Actuators, B* **107**, 936–944 (2005).
2. Martins, V. C. *et al.* Femtomolar limit of detection with a magnetoresistive biochip. *Biosens. Bioelectron.* **24**, 2690–2695 (2009).
3. Gaster, R. S. *et al.* Matrix-insensitive protein assays push the limits of biosensors in medicine. *Nat. Med.* **15**, 1327–1332 (2009).
4. Gaster, R. S. *et al.* Quantification of protein interactions and solution transport using high-density GMR sensor arrays. *Nat. Nanotechnol.* **6**, 314–320 (2011).
5. Henriksen, A. D., Wang, S. X. & Hansen, M. F. On the importance of sensor height variation for detection of magnetic labels by magnetoresistive sensors. *Sci. Rep*, accepted (2015).
